# Increased COVID-19 infection risk, COVID-19 vaccine inaccessibility, and unacceptability: Worrisome trio for patients with substance abuse disorders

**DOI:** 10.7189/jogh.11.03106

**Published:** 2021-10-02

**Authors:** Farah Yasmin, Hala Najeeb, Muhammad Sohaib Asghar, Irfan Ullah, Sheikh Mohammed Shariful Islam

**Affiliations:** 1 Department of Internal Medicine, Dow Medical College, Dow University of Health Sciences, Karachi, Pakistan; 2 Department of Internal Medicine, Dow University Hospital – Ojha Campus, Dow University of Health Sciences, Karachi, Pakistan; 3 Department of Internal Medicine, Kabir Medical College, Gandhara University, Peshawar, Pakistan; 4 Institute for Physical Activity and Nutrition, School of Exercise and Nutrition Sciences, Faculty of Health, Deakin University, Burwood, Australia

The coronavirus 2019 (COVID-19) pandemic has massively strained the health care system and the lives of individuals, causing mass destruction to social and economic structures. The greatest impact, however, is probable in the stigmatized community that suffers from substance use disorders (SUDs) including opioid, and alcohol use disorders (AUD) [1].

## INCREASED COVID-19 INFECTION SEVERITY AMONG SUD PATIENTS

SARS-COV-2, aided by its spike protein, binds to angiotensin-converting enzyme-2 (ACE-2) receptors, releasing its genomic mRNA. Vascular endothelial cells express ACE-2 receptors in the heart, kidney, and lungs. This increased expression leads to endotheliitis and hence, a prognosis of organ damage in COVID-19 [1]. Studies have proved that comorbidities like COPD, heart disease, and hypertension increase the risk of COVID-19 infection. People who excessively use drugs such as opioids, marijuana, tobacco, nicotine, and alcohol suffer from similar diseases and are more likely to contract COVID-19 [2]. Increased expression of ACE-2 receptors in chronic illnesses facilitates the entry of the virus. Moreover, such individuals suffer from a worse prognosis of coronavirus as the cytokine storm leads to hyperinflammation [3].

The clash of the opioid epidemic with the ongoing pandemic has led to a 46% prevalence of COVID-19 cases. Hypoxemia and cardiomyopathy manifest in individuals who overdose on methamphetamine (METH), and opioids including the heroine, morphine, and fentanyl [4,5]. Recreational drugs such as cocaine and marijuana suppress T-cell activation in response to antibodies thereby reducing SARS-CoV-2 clearance. Cocaine, marijuana, and amphetamines alter the hypothalamus-pituitary-axis that regulates pathogens in the body. Thus, immunosuppression of macrophages, T-cells, and B-cells puts the individuals at greater risk of contracting COVID-19 [1]. Overuse of alcohol, METH, and cocaine decreases the expression and degrades membrane proteins such as tight junctions. The blood-brain barrier (BBB) loses its integrity and undergoes cytoskeletal remodeling. With the increased permeability of BBB, neuroinflammation and SARS-CoV-2 invasion can lead to brain edema and degeneration [1,6]. Components of synthetic cannabinoids and marijuana activate CB1R in the brain, and cannabinoid receptors in the myocardium, vascular endothelial cells, and smooth muscles of the heart. This results in stroke, COPD, and arrhythmia which are associated with predisposing risk factors of COVID-19 [1].

**Figure:**
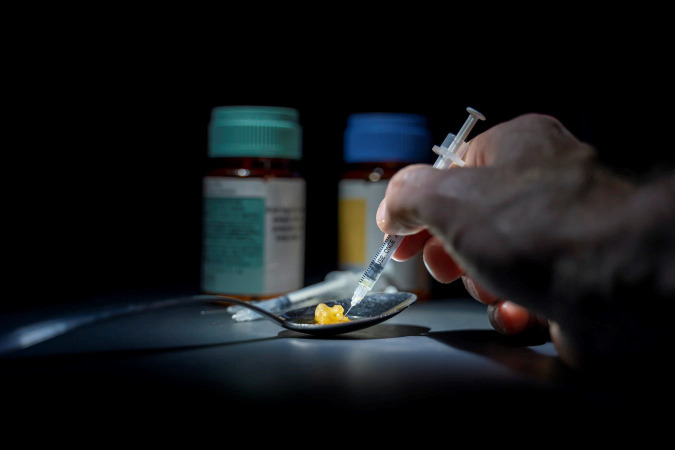
Photo: COVID-19 vaccine uptake in substance use disorder patients is challenged by the structural and financial barriers to vaccination centres (Michael Longmire, via unsplash.com).

AUD also increases the release of inflammatory cytokines such as TNF-α, which is a key contributor to the SARS-CoV-2 infection [7]. Immunosuppression is observed in 70% of AUD and alcoholic liver disease cases. This might lead to HIV and put patients suffering from this lethal combination at a three-times higher risk of COVID-19 [8,9]. Furthermore, substance abuse doubles the risk of community-acquired pneumonia, paving the path for SARS-CoV-2. Dyspnea and reduced lung capacity are hallmarks of COVID-19 infection, which are more commonly manifested in individuals with SUDs, and comorbid cardiopulmonary infections.

Treatment for people who abuse drugs includes opioid agonists such as methadone, buprenorphine, and take-home naloxone. Reduced staff, quarantine, and reduced visits to the clinics during the pandemic can trigger anxiety, force self-isolation, and increase the chances of overdosing. The risk of relapse is bound to multiply in SUD individuals who are under remission, especially when securing treatment services becomes difficult. Owing to the vulnerability and susceptibility to SARS-CoV-2 in such individuals, this jeopardizes any progress made [10].

## INCREASED COVID-19 INFECTION RISK, AND VACCINE UNACCEPTABILITY AMONG SUD PATIENTS

Pre-existing pathological disturbances in SUD individuals are affected by the behavioral challenges and societal norms that are attached to COVID-19. While preventive measures and therapeutic strategies reduce mortality rates, the long-term solution of adverse COVID-19 implications is vaccination. With the advent of efficacious COVID-19 vaccines, the key challenge is overcoming vaccine hesitancy [11].

A cross-sectional study in the U.S has shown decreased willingness to be vaccinated against COVID-19 in the vulnerable population that overdoses on cannabis [12]. Another study in the U.S showed that 52.3% of adults who used marijuana, and 55.6% of adults who heavily drank at least once in the past 30 days were indecisive or unwilling to receive the COVID-19 vaccine [13]. Low rates of literacy and awareness can lead to misinterpretation of COVID-19 symptoms in opiate users as signs of withdrawal, which leads to further dosing of opioids [10]. Lack of knowledge in these groups against COVID-19, its severity, rapid vaccine developments, and myths has raised doubts about the safety and efficacy of the vaccine among them [11].

People who excessively use harmful drugs are confined to open-drug prisons, housing instability, and small spaces. COVID-19 preventive measures are hence the least observed in such groups. Repetitive spitting habits, sharing needles, and hookahs make them the greatest disseminators of the virus [14]. A compromised blood-brain barrier and respiratory toxicity in SUDs with COVID-19 puts them on the vaccination priority list.

It is imperative to understand that a global health issue cannot be curtailed without vaccinating the high-risk population. Awareness programs can reduce vaccine hesitancy globally. However, in low- and middle-income countries where government funding is low, this challenging task can be aided by charitable organizations. Trained volunteers can continue to visit less-privileged areas regularly to distribute protective equipment and sleeping bags, and to demonstrate preventative measures.

## COVID-19 VACCINE INACCESSIBILITY AMONG SUD PATIENTS

Reduced rates of vaccination have been further challenged by vaccine distribution plans. Initial vaccine distribution was limited to the health care workers and older population, with people who overdose on drugs remaining out of the picture [14]. The attempt to eradicate a deadly disease that has taken every corner of the world is based on global inequity [15]. Vaccine procurement by the privileged and the pre-existing barriers have usurped the freedom and right to vaccination of challenged individuals with SUDs.

In addition to this is the fact that families showing an addiction pattern are dependent on a sole earner. The economic instability during the pandemic has shifted priorities from health care to procuring housing, food, and drug supply. With the ongoing pandemic magnifying unemployment in an already unstable population, a stronger financial net by the local municipal should cover free and safe shelter, and consistent food supplies as incentives for COVID-19 vaccines.

The financial barrier is further widened with limited access to technology. This has decreased vaccine registrations, accessibility and locating vaccination sites, or tracking of patients suffering from SUDs. Misinformation about the vaccine eligibility criteria proves to be an additional barrier [16]. The revolutionary practice of telehealth has allowed few caregivers to connect with their patients. Access to technology at addiction care centers, or temporary housing in hotels can be an incentive for overdosing individuals to get vaccinated. In absence of direct care from physicians, digital applications are being used to track vaccine signups, withdrawal symptoms and extend therapy through videocalls, as in British Columbia [17]. However, implementation of digital strategies is difficult in developing countries where drug abusers often do not have access to Wi-Fi.

Unavailability of low-cost transportation services to vaccination centers is another obstacle. Free transportation services and attendings for high-risk SUD patients should be provided. Since mobility remains an issue, multiple walk-in vaccination sites can be set up at methadone clinics, syringe services programs, Alcoholics Anonymous, and Narcotics Anonymous meetings to amplify vaccination rates [18].

Moreover, as health care insurance remains a far-fetched idea for them, the cost incurred is a key factor that is considered for COVID-19 vaccinations and screening. Developing countries have limited insurance programs, and an infrastructure that does not support vaccination sites. Many find themselves in debts that originate from persistent dependency and the purchase of drugs [19,20]. As AUD and SUDs are common in undocumented immigrants, the perpetual fear of the law limits their interaction with the health care system.

## STRATEGIES TO MITIGATE THE DISPARITY IN COVID-19 VACCINATION, AND REDUCE COVID-19 INFECTION RISK AMONG SUD PATIENTS

Previous prominent pandemics as the Ebola, Spanish Flu, or Zika Virus were sustained with host immunization. Therefore, an effective global approach is required by each country. Low- and middle-income countries (LMIC) with ineffective and out-stretched health care systems are likely to not have records of individuals suffering from SUDs. The following strategies could be employed in reducing COVID-19 infection risk and improving vaccination rates among patients suffering from SUDs as listed below, and in **Table 1**.

**Table 1:** Barriers and solutions to COVID-19 vaccine access for people with substance abuse disorders

Barriers	Solutions/policies
**Vaccine awareness and education**	COVID-19 vaccination awareness programs to debunk myths, address vaccine safety, and efficacy.
	Educate regarding the severity of SARS-CoV-2 in individuals with comorbidities.
	Spreading awareness about COVID-19 preventive measures, including social distancing.
	Teaching the use of technology to individuals to tackle telehealth opportunities.
**Engagement with vaccination programs**	Training a trusted workforce to administer COVID-19 vaccines
	Providing incentives at vaccination sites, including food coupons, and free housing
	Arranging transportation services to vaccination sites and providing an attending for at-risk patients
	Hassle-free, walk-in vaccination service for SUDs individuals
**Peer support, training and resources**	Forming a close-knit peer network of SUD individuals and those in remission, to target social isolation in the pandemic Encouraging vaccine receivers to share their experience to reduce stigma
**Organizational resources for a vaccine program**	Local outreach teams of social workers to diagnose, and identify SUD individuals
	Setting up vaccine sites in methadone, and opioid treatment centers
	Arranging temperature-regulated sites for COVID-19 vaccine storage
**Finances**	The government allocated funds to pay for the vaccines
	Subsidies on health insurance to encourage further vaccine sign ups
**Policies**	Prioritizing vaccine allocation to the high-risk population
	Procurement and administering single-dose COVID-19 vaccines to drug addicts
	Allowing the provision of take-home treatment services

SUD – substance use disorder

An important initial step in LMICs includes devising a localized program with public health workers and psychiatrists to reach out and diagnose individuals with SUDs. These outreach programs will help address the perception against health care workers, and COVID-19 vaccines [14].

1. Adopting unified addiction care services and increasing the workforce will mitigate psychological effects in individuals with SUDs as they will receive treatment in a non-discriminatory environment [14].
2. Support groups for diagnosed SUDs, as in Oregon, will help fight social isolation. In LMICs particularly where peer-support networks are rare, a tightly knit community can be promoted though communal activities. Vaccinated individuals can share experiences to help destigmatize the vaccination drive.
3. Maintaining balance and coordinating daily tasks is a challenging task for the high-risk population. Promising a long-term strongly knit primary health care service with transportation and attendings for them can improve turnovers at clinics for check-ups and vaccinations [14,21]. A trusted workforce can be trained to introduce vaccines to individuals in remission, or treatment.
4. Local governments could opt for single-dose vaccines, owing to an unwavering turnout for the second dose.

Sweden’s National Board of Health and Welfare and the Substance Abuse and Mental Health Services Administration in the United States released guidelines for safer methadone administration in individuals enrolled in opioid treatment programs [21]. Buprenorphine, a drug for opioid disorders, could not be prescribed in the initial COVID-19 wave with the closure of care centers. Recent guidelines by the Drug Enforcement Agency have allowed its prescription by physicians over the phone [22]. An initiative by the Foundation for Opioid Response Efforts (FORE) and the Addiction Policy Forum (APF) has allocated funds to recruit ‘vaccine navigators’ to tackle vaccine hesitancy [16]. United Nations 2030s agenda of a sustainable world emphasizes narcotics abuse prevention methods [23]. The overly debated notion of providing a ‘safe drug supply’ to limit the overdosing crises in the pandemic received attention from the Canadian Association of People who Use Drugs (CAPUD) which modelled supply plans [24]. As the risks of COVID-19 infections intensify, these nation-centric policies to tackle the crisis seem insufficient. Thus, unified proactive guidelines to tackle the COVID-19 and substance abuse syndemic need to be devised into policies by global health forums and mandated to be adopted by each country.
